# Prevalence, Risk Factors, and Management of Prehypertension

**DOI:** 10.4061/2011/605359

**Published:** 2011-10-29

**Authors:** Wenwen Zhang, Ninghua Li

**Affiliations:** Department of Epidemiology, Beijing Hospital and Beijing Institute of Geriatrics, Key Laboratory of Geriatrics, Ministry of Health, Beijing 100730, China

## Abstract

Prehypertension remains an important public health challenge all over the world and appropriate treatments should be adopted to prehypertensive group in different degree effectively. This review aimed to assess the prevalence of Prehypertension and provide effective evidence of the benefits of treating prehypertensive patients. The reasonable evaluation and appropriate intervention of prehypertensive remain need further study.

## 1. Introduction

Prehypertension was defined as a systolic blood pressure of 120–139 mmHg and/or a diastolic blood pressure of 80–89 mmHg. The concept of prehypertension was introduced as the new guideline for the management of blood pressure by the seventh report of the Joint National Committee on Prevention, Detection, Evaluation, and Treatment of High Blood Pressure (JnC-7) [1]. The objectives of defining this classification of blood pressure were to draw the clinical and public healthy attention on the prevention of people in this range. Prehypertension is a precursor of clinical hypertension and is closely related with the increased incidence of cardiovascular disease [2–4]. Patients with Prehypertension (120–139/80–89 mmHg) have an increased risk of cardiovascular morbidity and mortality compared with patients who have normal blood pressure (<120/80 mmHg). This paper aimed to assess the prevalence of Prehypertension and provide effective evidence of the benefits of treating prehypertensive patients in community.

## 2. Epidemiology

### 2.1. High Prevalence of Prehypertension

The National Health and Nutrition Examination Survey (NHANES) 1999-2000 reported that the overall prevalence of prehypertension was 31% all over the world, which was higher in men than in women [5]. A statistical analysis of disease-free adult NHANES participants which was conducted from 1999 to 2006 found that the overall prevalence of PreHTN in disease-free adults was 36.3% [6]. The ATTICA study which included 1514 men and 1528 women found that the prehypertensive population was 39% (43% in men and 35% in women) [7]. The prevalence of prehypertension in India was found more than 45% (of the 2,007 people studied, 47.4% had Prehypertension and 34.7% had hypertension. Prehypertension was found in 46.6% of the men and 49.8% of the women). The data from Korean Nation Health and Nutrition Survey 2001 reported that the estimated age-adjusted prevalence of hypertension and prehypertension was 22.9% (26.9% in men, 20.5% in women) and 31.6% (41.9% in men, 25.9% in women [8]). The Jichi Medical School Cohort Study showed that the prevalence of prehypertension was 34.8% (males) and 31.8% (females) in Japanese general population [9]. Cross-sectional surveys of Shandong and Wuhan Provinces revealed that the prevalence of prehypertension was more than 40% in China [10, 11].

### 2.2. Risk Factors

 Prehypertension is correlated with the recognized traditional cardiovascular risk factors such as obesity, diabetes mellitus, and dyslipidemia. NHANES II 1999-2000 data showed that 64% of individuals with prehypertension had at least another cardiovascular risk factor; persons with Prehypertension were 1.65 times more likely to have at least another adverse risk factor than those with normotension and the percentage increased to 94% in those aged 60 years or older [12]. Another mortality study of adults aged 30–74 years at the time of the NHANES II examination showed that almost 90% of individuals with prehypertension had at least one other cardiovascular risk factor [13]. Many studies demonstrated that the prehypertensive group had higher levels of blood glucose, total cholesterol, low-density lipoprotein cholesterol, and triglycerides, higher body mass index, and lower levels of high-density lipoprotein cholesterol than the normotensive group [14, 15]. Obesity, abnormalities of glucose metabolism, and insulin resistance were the major factors associated with prehypertension and hypertension [16]. BMI was a strong predictor of prehypertension. The Jichi Medical School Cohort Study which enrolled 4,706 males and 7,342 females of Japanese general population suggested that body mass index (BMI) of more than 23.0 kg/m^2^ was the strongest determinant of prehypertension [9]. Prehypertension was more prevalent in diabetic than nondiabetic participants. Compared with nondiabetic participants with normal blood pressure, the hazard ratios of cardiovascular disease were higher for those with both prehypertension and diabetes than for those with prehypertension alone [17].

Other nontraditional cardiovascular risk factors had also been relevant to the development of prehypertension. The prevalence of metabolic syndrome in the prehypertension group was higher than in the normal BP group. Larger waist circumference and body mass index, higher levels of triglycerides, fasting blood glucose, uric acid and ferritin, and lower levels of high-density lipoprotein-cholesterol were more common in subjects with prehypertension than in those with normal BP [18]. Compared to normotensives, prehypertension had higher C-reactive protein, tumor necrosis factor-alpha, amyloid-a, homocysteine levels, and higher white blood cell counts after correcting for multiple comparisons and adjusting for age, body mass index, blood lipids, glucose, food groups consumed, and other potential confounders [19]. It was also found that the prevalence of microalbuminuria in the prehypertension group was higher than in the normal BP group [18]. The nationally representative sample of US adults among 5,827 participants without cardiovascular disease (CVD) and hypertension concluded that prehypertension was associated with higher serum gamma-glutamyltransferase (GGT) levels [20]. In recent years, people pay more attention to whether prehypertension causes change of myocardial structure and function. After adjusting for intergroup differences in age, diabetes, body mass index, smoking, study center, and plasma creatinine, and mean values for left ventricular (LV) measurements were found to be significantly increased in both the prehypertensive and hypertensive groups compared with the normal blood pressure group. LV systolic and diastolic function differed significantly in the hypertensive groups, but not in the prehypertensive participants, compared with the normal blood pressure group [21]. It suggested that the LV structure had been changed during prehypertension period; however, the LV systolic and diastolic function had not been impacted by the change.

### 2.3. Cardiovascular Disease and Prehypertension

 Individuals with prehypertensive levels of blood pressure had an increased risk of developing cardiovascular disease relative to those with optimal levels. The association was pronounced among individuals with diabetes mellitus, and among those with high BMI [22]. It was also found that prehypertension was associated with an increased risk for cardiovascular disease, including myocardial infarction (MI) and coronary artery disease (CAD), but no stroke, with a mean follow-up period of 10 years [23]. The Jichi Medical School Cohort Studys of Japan discovered that prehypertension was associated with a 45% higher risk of cardiovascular events than normal blood pressure after adjusting for traditional cardiovascular risk factors. Prehypertension was associated with an increased 10-year risk of cardiovascular disease; the risk of cardiovascular events with prehypertension during the second 5-year period was elevated in the nonelderly subgroup (<65 years) [9]. A meta-analysis that included approximately 1 million individuals from 61 long-term epidemiological studies demonstrated that mortality from ischemic heart disease and stroke in individuals aged 40–89 years increased in a log-linear relationship together with increases in both systolic/diastolic blood pressure. For each 20 mmHg increase in systolic blood pressure or 10 mmHg increase in diastolic blood pressure over 115/75 mmHg, there was a twofold increase in mortality associated with coronary artery disease and stroke [24]. Longitudinal data from the Framingham Heart study indicated that individuals formerly classified as having “normal” and “high-normal” blood pressure (120–139/80–89 mmHg) were at increased risk of developing full-blown hypertension and cardiovascular disease later in life than those who had an optimal blood pressure (<120/80 mmHg) [25]. The study among 68,438 urban Chinese women aged 40–70 years during an average of 5 years of followup showed that hypertension was associated with high stroke mortality [26].

## 3. Treatment

### 3.1. Strategy of Treatment

The relationship between prehypertension and cardiovascular disease aroused widespread concern, and it became an important subject to prevent and intervene. JNC-7 suggested the individuals of prehypertension adopted a healthy lifestyle in order to lower blood pressure and prevent progression to hypertension, with associated reductions in target organ damage and cardiovascular events. 2007 ESH-ESC Practice Guidelines for the Management of Arterial Hypertension emphasized that cardiovascular complications of patients should immediately take drug therapy to reduce the risk of cardiovascular events even in the scope of prehypertensive [27].

### 3.2. Nonpharmacological Treatments

Prehypertensive blood pressure levels identify individuals with elevated risk of developing hypertension. Prehypertensive patients are not the usual candidates for antihypertensive drug therapy, and prehypertensive individuals should primarily be advised to modify their lifestyle to lower their blood pressure to normal values (systolic/diastolic blood pressure <120/80 mmHg) to reduce the risk of developing hypertension. Lifestyle modifications were the main treatment recommended by JNC-7 guidelines for the prehypertension patients. The lifestyle modifications included (1) Lose weight, maintain normal body weight, keep body mass index between 18.5 and 24.9 kg/m^2^. (2) Adopt DASH eating plan, consume a diet rich in fruits, vegetables, and low-fat dairy products with a reduced content of saturated and total fat. (3) Adopt dietary sodium reduction and reduce dietary sodium intake no more than 100 mmol per day (2.4 g sodium or 6 g sodium chloride). (4) Promote physical activity. (5) Attempt moderation of alcohol.

### 3.3. Dietary Approaches

It is known that obesity, sodium intake, and alcohol consumption factors influence blood pressure. The DASH diet is recommended by physicians for people with hypertension (high blood pressure) or prehypertension. The DASH diet eating plan has been proven to lower blood pressure in studies sponsored by the National Institutes of Health (Dietary Approaches to Stop Hypertension). In addition to being a low-salt (or low-sodium) plan, the DASH diet provides additional benefits to reduce blood pressure. It is based on an eating plan rich in fruits and vegetables, and low-fat or nonfat dairy. DASH dietary pattern is rich in potassium (from fruits and vegetables) and calcium (from dairy), low in total and saturated fat, and contains limited amounts of meats and sweets [28]. Compared with a typical American control diet, the DASH dietary pattern reduced SBP by 5.5 mmHg and DBP by 3.0 mmHg overall. In the participants with prehypertension, corresponding reductions were 3.5 mmHg and 2.1 mmHg. It was also found that the reduction of sodium intake levels below the current recommendation of 100 mmol per day and the DASH diet both lower blood pressure substantially, with greater effects in combination than singly. Long-term health benefits will depend on the ability of people to make long-lasting dietary changes and the increased availability of lower-sodium foods [29].

### 3.4. Weight Reduction

Increased body weight is a strong risk factor for prehypertension and weight loss is important for the prevention and treatment of prehypertension and hypertension. A lot of clinical trial data document the significant BP-lowering effect of weight loss. A meta-analysis of randomized controlled trials included twenty-five randomized, controlled trials (comprising 34 strata) published between 1966 and 2002 with a total of 4874 participants was performed to estimate the effect of weight reduction on blood pressure overall and in population subgroups in 2003. Blood pressure reductions were −1.05 mmHg (95% CI, −1.43 to −0.66) systolic and −0.92 mmHg (95% CI, −1.28 to −0.55) diastolic when expressed per kilogram of weight loss in this study. It was found that physical exercise with weight reduction reduced blood pressure, decreased cardiovascular risks, and improved abnormal left ventricular relaxation recently [31].

### 3.5. Salt Intake Reduction

Many surveys reveal the consistent correlation between sodium intake and BP. Numerous trials show that the limit of sodium intake leads to reductions in BP [32, 33]. A long-term followup assessed 10–15 years after the original trial at 10 clinic sites in 1987–90 (TOHP I) and nine sites in 1990–5 (TOHP II) showed the remote effects of dietary sodium reduction for 18 months (TOHP I) or for 36–48 months (TOHP II) on risk of cardiovascular disease. Risk of a cardiovascular event was 25% lower among those in the intervention group compared with the matched group after adjusted for trial, clinic, age, race, and sex, and 30% lower after further adjustment for baseline sodium excretion and weight [34]. The study demonstrated that sodium reduction could reduce long-term risk of cardiovascular events in patients with prehypertension. However, it is difficult to maintain the reduction of sodium intake in the general public.

### 3.6. Physical Activity

 The correlation between habitual physical activity and the development of hypertension have been found in numerous studies. A meta-analysis demonstrated the studies published and indexed between January 1966 and December 1998 concluded that progressive resistance exercise was efficacious for reducing resting systolic and diastolic blood pressure in adults [35]. Another Meta-analysis of randomized, controlled trials evaluated the effect of aerobic exercise on blood pressure. In the random-effect model, it was found that aerobic exercise was associated with a significant reduction in mean systolic and diastolic blood pressure 3.84 mmHg and 2.58 mmHg, respectively [36]. A study evaluated appropriate type and frequency of physical activity for the beneficial effect on blood pressure among Japanese male workers. There was a progressive reduction in the hazards ratios of hypertension with increasing total daily activity (hazards ratio of 0.65 in subjects who walked >8000 steps/day versus <4000 steps/day). Subjects who exercised >3 times/week also showed a significantly lower risk (0.35) of developing hypertension versus those who exercised <3 times/week. In addition, accumulating intermittent bouts of physical activity, as short as 10 min, total 30 min walk sessions may reduce systolic BP in prehypertension.

### 3.7. Moderation of Alcohol

The recommendation of JNC-7 suggested that the limit consumption to no more than 2 drinks (1 oz or 30 mL ethanol; e.g., 24 oz beer, 10 oz wine, or 3 oz 80-proof whiskey) per day in most men and to no more than 1 drink per day in women and lighter weight persons. The regular consumption of alcohol elevates blood pressure and the global estimates showed that the attributable risk for hypertensive disease from alcohol was 16%. The increase of blood pressure is approximately 1 mmHg for each 10 g alcohol consumed and is largely reversible within 2–4 weeks of abstinence or a substantial reduction in alcohol intake, and this increase of blood pressure occurs irrespective of the type of alcoholic beverage. Maximum cardiovascular benefit occurs at relatively low levels of consumption (i.e., one to two standard drinks a day in men (10–20 g alcohol) and up to one a day in women (10 g alcohol)). In hypertensive subjects, consumption beyond these levels would be unwise [37].

The reduction of blood pressure is always the comprehensive effect of the lifestyle interventions in the management of prehypertension. The main 6-month results from the PREMIER trial showed that comprehensive behavioral intervention programs improved blood pressure [38].

### 3.8. Pharmacological Treatments

Whether people without diabetes or chronic kidney disease (CKD) should be given pharmacological treatments or not is still on discussion [39]. The pharmacological treatments could be used on condition that lifestyle modification trial fails to reduce blood pressure to 130/80 mmHg or less according to the JNC-7 guidelines for prehypertension without diabetes or chronic kidney disease (CKD) [1]. The trial of TROPHY study evaluated the effect of the angiotensin II receptor antagonist candesartan cilexetil on the prevention of transition from prehypertension to stage 1 hypertension [40]. Participants were randomly assigned to receive two years of candesartan (Atacand, AstraZeneca) or placebo, followed by two years of placebo for all. When a participant reached the study end point of stage 1 hypertension, treatment with antihypertensive agents was initiated. Both the candesartan group and the placebo group were instructed to make changes in lifestyle to reduce blood pressure throughout the trial. Over a period of four years, stage 1 hypertension developed in nearly two thirds of patients with untreated prehypertension, and the prehypertension treated with candesartan appeared to be well tolerated and reduced the risk of incident hypertension during the study period. Another study evaluated the impact of bovine casein hydrolysate (c12 Peptide) on prehypertension. After four weeks, repeated daily intake of 3.8 g C12 peptide, the systolic, and diastolic BP reduced significantly by about 10 mmHg and 7 mmHg, respectively [41]. The drug intervention in patients with prehypertension is therefore appealing. In the absence of higher baseline risk, the absolute benefit of treatment is presumably small and was not demonstrated to date. These individuals could be candidates to treatment with the aim to prevent the development of full hypertension. The long-lasting effectiveness of nondrug therapies is low outside the controlled conditions of randomized clinical trials, and there is evidences that the use of BP-lowering drugs reduces the incidence of hypertension in individuals with prehypertension by more than 60%. Clinical trials testing the efficacy and safety of BP agents to prevent hypertension in a population-based perspective are required. In the meantime, it is worthy to present the option to start low doses of BP agents for individuals with prehypertension without comorbidities who do not respond to the prescription of lifestyle modification [42].

## 4. Conclusions

The category of “prehypertension” increases the awareness of the high risk group of hypertension. Individuals with Prehypertension have an increased risk of full-blown hypertension, target organ damage, and cardiovascular-related morbidity and mortality [43]. Despite progress in recent years in the prevention, detection, and treatment of Prehypertension, it remains an important public health challenge that adopts appropriate treatments to prehypertensive group in different degrees effectively. The reasonable evaluation and appropriate intervention of prehypertensive need further study.
